# Assessing the potential for Bluetongue virus 8 to spread and vaccination strategies in Scotland

**DOI:** 10.1038/srep38940

**Published:** 2016-12-13

**Authors:** Paul R. Bessell, Kate R. Searle, Harriet K. Auty, Ian G. Handel, Bethan V. Purse, B. Mark de C. Bronsvoort

**Affiliations:** 1 The Roslin Institute, The University of Edinburgh, Easter Bush, EH25 9RG, UK; 2 Centre for Ecology and Hydrology, Edinburgh, EH26 0QB, UK; 3 Epidemiology Research Unit, Future Farming Systems Group, Scotland’s Rural College (SRUC), An Lòchran, Inverness Campus, Inverness, IV2 5NA, UK; 4 Royal (Dick) School of Veterinary Studies, The University of Edinburgh, Easter Bush, EH25 9RG, UK; 5 Centre for Ecology and Hydrology, Wallingford, OX10 8BB, UK

## Abstract

Europe has seen frequent outbreaks of Bluetongue (BT) disease since 2006, including an outbreak of BT virus serotype 8 in central France during 2015 that has continued to spread in Europe during 2016. Thus, assessing the potential for BTv-8 spread and determining the optimal deployment of vaccination is critical for contingency planning. We developed a spatially explicit mathematical model of BTv-8 spread in Scotland and explored the sensitivity of transmission to key disease spread parameters for which detailed empirical data is lacking. With parameters at mean values, there is little spread of BTv-8 in Scotland. However, under a “worst case” but still feasible scenario with parameters at the limits of their ranges and temperatures 1 °C warmer than the mean, we find extensive spread with 203,000 sheep infected given virus introduction to the south of Scotland between mid-May and mid-June. Strategically targeted vaccine interventions can greatly reduce BT spread. Specifically, despite BT having most clinical impact in sheep, we show that vaccination can have the greatest impact on reducing BTv infections in sheep when administered to cattle, which has implications for disease control policy.

Between 2006 and 2008, north-west Europe experienced annual outbreaks of Bluetongue virus (BTv) serotype 8, which caused significant impact on livestock industries. BTv is vectored by *Culicoides spp.* and can infect multiple ruminant species. Sheep are of highest importance in terms of the clinical impact of bluetongue disease (BT)^1^,^2^. Cattle are also susceptible but remain largely asymptomatic, although some studies have reported clinical disease in cattle during BTv-8 outbreaks^3^,^4^. Cattle are however an important reservoir species for infection^5^, but susceptible wildlife species such as deer may play an important role in maintaining transmission^6^,^7^.

There were multiple incursions of BT into southern Europe between 1998 and 2005 and in 2006 BTv (serotype 8) was reported in northern Europe. BTv-8 successfully overwintered in 2006 and 2007 and spread widely during 2006, 2007 and 2008. It spread to the UK in 2007, with vaccine being used in disease control in 2008 in the UK^8^ and Europe more widely^2^. In Scotland, compulsory vaccination of cattle and sheep was implemented in 2008.

BTv-8 had not been reported in Europe between 2008 and 2015 but during the second half of 2015 an outbreak was reported in central France^6^ that continued into 2016. Given the distribution of BTv-8 in France in the winter of 2015/2016, the risk of introduction to the UK during 2016 was estimated to be high^9^.

In temperate latitudes, the spread of BT and many other arboviruses is seasonally limited, with a seasonal vector free period in winter when temperatures are too low for within-vector viral replication and for adult vector activity^10^,^11^. The seasonal limitations to spread are particularly pertinent to Scotland^12^,^13^,^14^,^15^. Consequently, different scenarios of disease incursion in terms of seasonal timing and seasonal temperature conditions could produce a wide range of potential outcomes. Understanding the potential impact of the disease under a range of scenarios is therefore critical to enable realistic contingency planning. Whilst temperatures are believed to be important to the epidemiology of BTv in Scotland, rainfall may be less of a constraint in this region of high rainfall. Rainfall effects BTV transmission by governing the availability of semi-aquatic breeding sites for immature midges and watering areas for vertebrate hosts and by modulating survival and dispersal of adult midges^16^,^17^. However its interactive impacts with temperature on specific parameters of the transmission cycle and on dispersal processes are not well understood for any bluetongue episystem but particularly for Europe^2^. Consequently, we do not consider rainfall here.

The potential for BT to spread and the options for control in north-west Europe were considered during the BTv-8 outbreaks in 2006–2008^12^ and a number of incursion scenarios for Scotland were assessed^14^. However, recent developments have provided key information that warrants further assessment of incursion scenarios. These developments include: (i) better quantification of the extrinsic incubation period for BTv^11^; (ii) characterisation of the *Culicoides* vector populations in Scotland^10^,^18^,^19^,^20^; and (iii) demonstration of considerable host preference of the vector for cattle over sheep, such that when presented with a choice between feeding on a bovine or an ovine, between 80 and 90% of *Culicoides* that feed will take a blood meal from the bovine^21^,^22^. This may in part explain observations in France during the 2006–2008 BTv-8 outbreaks of considerably greater prevalences in cattle compared to sheep^6^. Whilst other serotypes of BTv have circulated in Europe in recent years, we focus on BTv-8 due to it’s history and wide geographical extent of spread in NW Europe.

To reassess the threat to Scotland and explore potential options for vaccination we use a published spatially explicit stochastic simulation model for Schmallenberg virus (SBV) spread in Scotland^13^,^15^ updated with parameters specific to BTv-8, and explore a number of scenarios for introduction and spread.

## Results

Here we present analysis of a number of different scenarios for BTv-8 spread in Scotland, exploring variations in the potential for spread due to intrinsic and extrinsic factors. Using this, we use a model of the basic reproduction number (R_0_) to explore the extent to which Scotland is suitable for BTv-8 transmission and then use a stochastic model to explore the range of spread under these scenarios as well as exploring the impacts of different strategies for vaccination against BTv-8.

### R_0_

**Figure 1:**
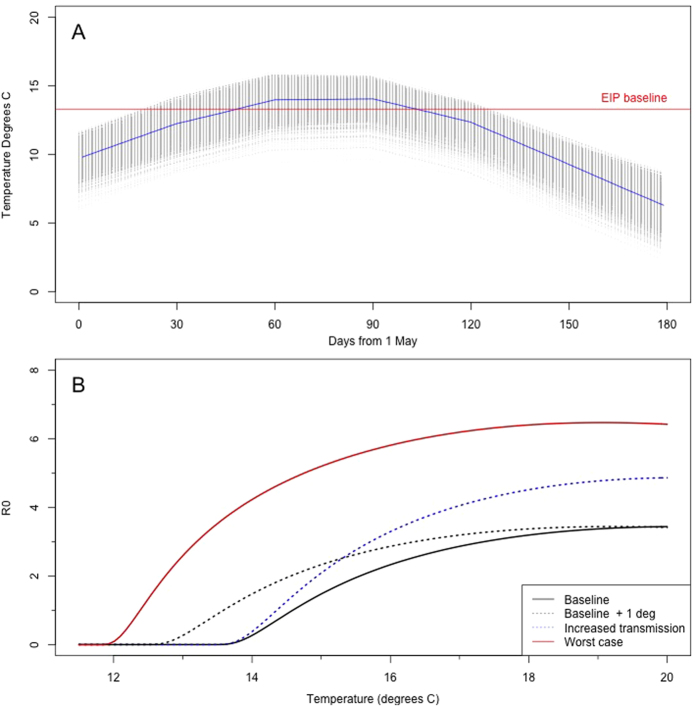
(**A**) The temperatures at different farms with the mean for all farms in Scotland (blue line) and the baseline temperature at which extrinsic incubation is possible (red line). (**B**) The R_0_ for different scenarios given the baseline temperature and adjustments to temperature and adjustment to extrinsic incubation temperature.

In terms of R_0_, Scotland is marginal for BT spread and only a proportion of farms are above the baseline temperature for the extrinsic incubation period (EIP) of 13.3 °C for some of the summer months (Fig. 1A). However, relatively small increases in temperature or reductions to the baseline temperature for EIP result in rapid increases to R_0_ (Fig. 1B).

**Figure 2:**
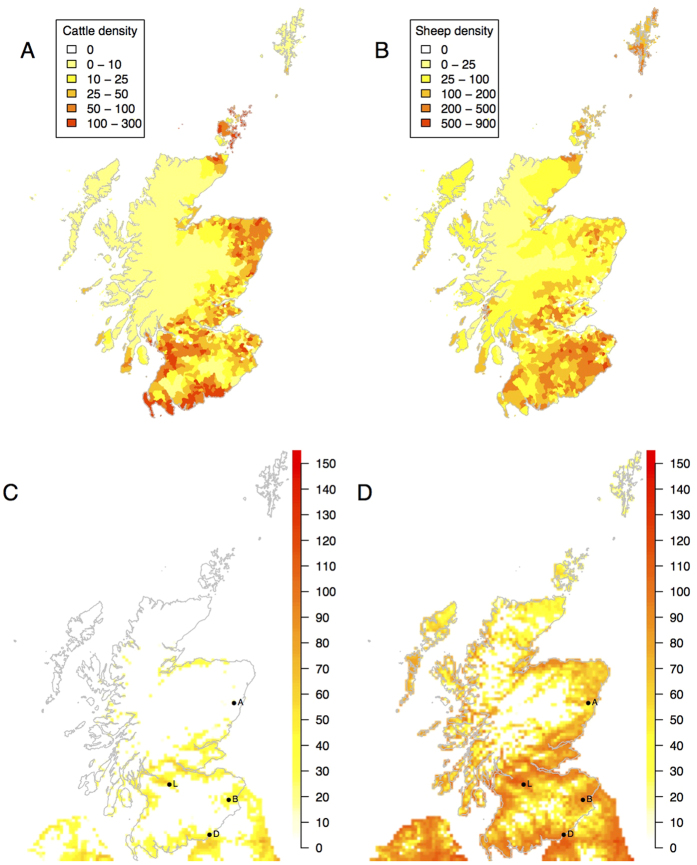
Density of cattle per km^2^ (**A**) and density of sheep per km^2^ (**B**) plotted by parish. The baseline (**C**) and worst case (**D**) scenario for BT in terms of potential for spread showing the number of days with an R_0_ greater than 1. Locations of the seeds are the black points. Maps created using R^45^.

The areas of Scotland that have the greatest number of days with an R_0_ greater than 1 are clustered in relatively small areas around southern areas and in a belt in the centre of the country between Edinburgh and Glasgow and south western coastal areas. This remains the case when the worst case scenario is considered, although in this scenario the areas are expanded, these areas that are suitable for transmission also correspond to areas of high livestock density (Fig. 2).

### BTv-8 spread scenarios

**Figure 3:**
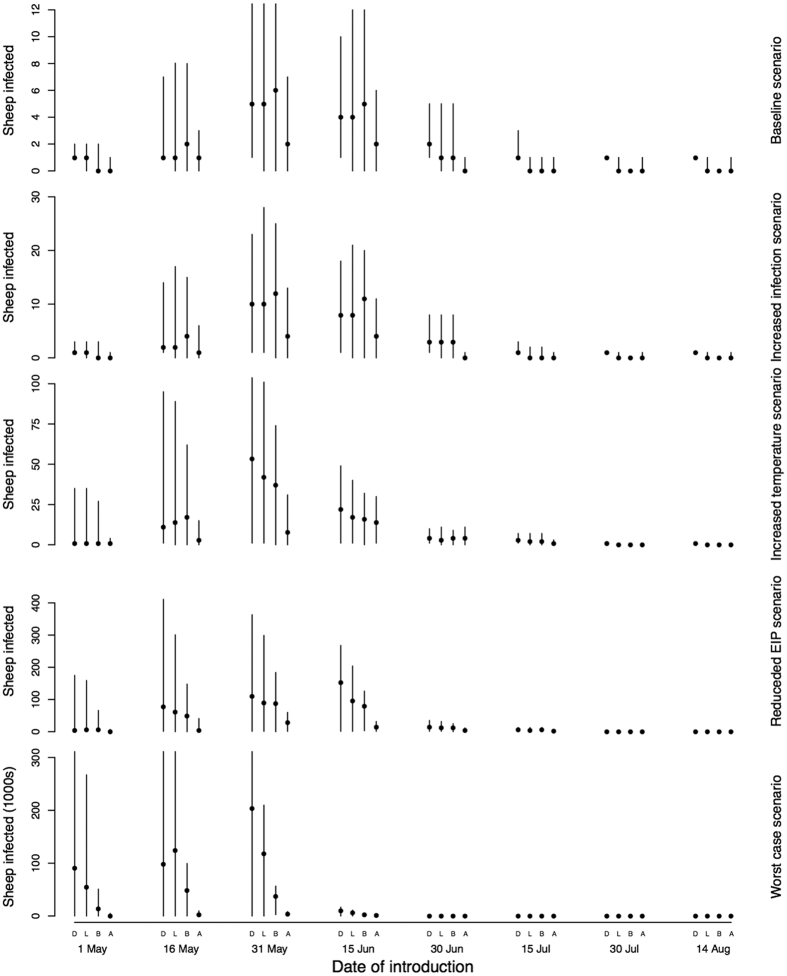
The number of sheep infected under different scenarios explored using this model, when incursion occurs at different times through the summer. The baseline parameter set (top), higher vector infection rate of 0.02 (second), temperature 1 °C higher (third), lower EIP base temperature of 12.7 °C and higher incubation rate of 0.026 (fourth) and a “worst case” scenario of all of these parameters (bottom). Note that the scale of the bottom (worst case) scenario is in 1000s. The points represent the medians and lines the 95 percentiles. The letters correspond to the sites of introduction: D to Dumfrieshire, L to Lanarkshire, B to the Borders, A to Angus.

Under the majority of scenarios, there is a relatively small amount of spread with fewer than 400 sheep typically infected following an individual incursion (Fig. 3). In the “worst case” scenario, several hundred thousand animals are infected and a large proportion of these are likely to show clinical signs, with up to 105,000 sheep deaths (Table 1). However, for this to occur, introduction must happen during a narrow window of optimal conditions for spread (Fig. 3).

### Vaccination

**Figure 4:**
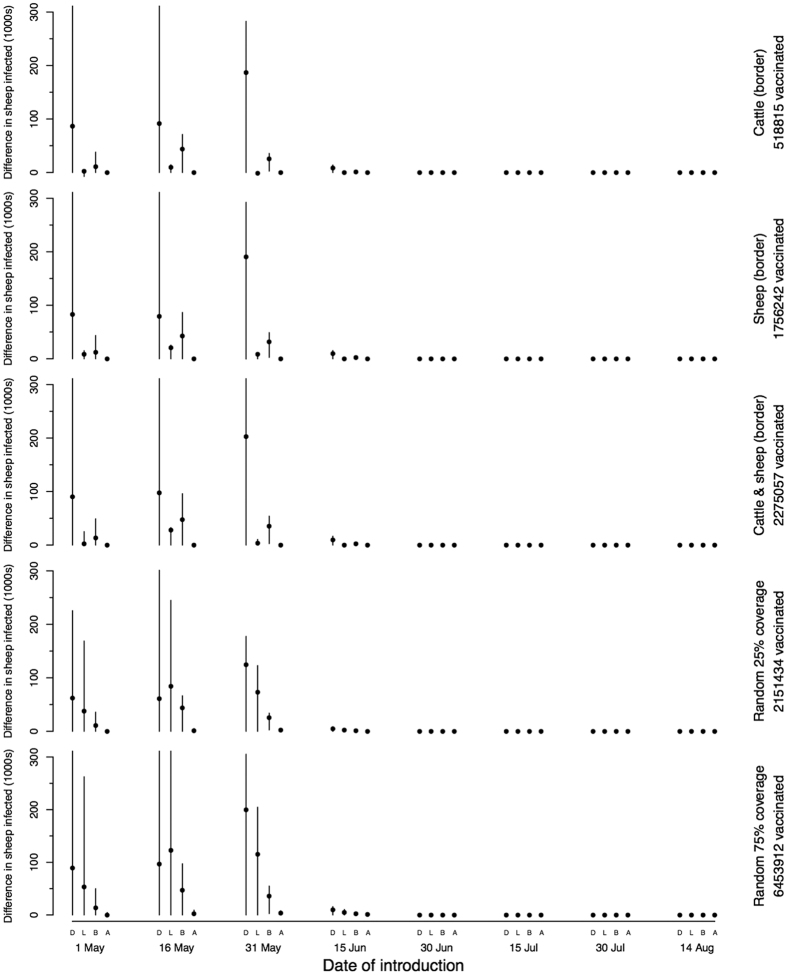
The reduction in the number of infected sheep when vaccination is applied in different scenarios under the worst case scenario described in Figs 2 and 4. Note that the y-axes are scaled in 1000s. The points represent the medians and lines the 95 percentiles.

Vaccination of cattle in the border area results in a mean difference of 186,000 sheep infected compared to the baseline and has the greatest impact when BTv is introduced into Dumfriesshire in southern Scotland (Fig. 4). There is a similar reduction in sheep infections when sheep in the border area are vaccinated, but this requires the vaccination of over 3 times the number of animals. However, following introduction to Lanarkshire, the same vaccination strategies confer little protection (Fig. 4, Table 1). Voluntary vaccination by 25% of farms results in a mean reduction in sheep infections of up to 123,000, but has substantial benefits irrespective of the site of introduction. Increasing from 25% to 75% the number of farms vaccinated by random vaccination offers potential benefits in terms of reducing sheep infections (Fig. 4) and reducing mortalities (Table 1), however, at 75% coverage, 6.45 million animals must be vaccinated. Increasing the extent of compulsory vaccination by vaccinating sheep and cattle in the borders area serves to further reduce the numbers of sheep deaths, but sheep deaths are reduced by up to 90% by vaccinating cattle alone (Table 1). When uptake of the vaccine is voluntary, the uptake needs to be very high to get a reduction in cases similar to vaccinating in just the south. Voluntary vaccination has a greater impact when disease is introduced elsewhere–in particular Lanarkshire (central Scotland) where strategic vaccination has only a small impact (Fig. 4).

**Figure 5:**
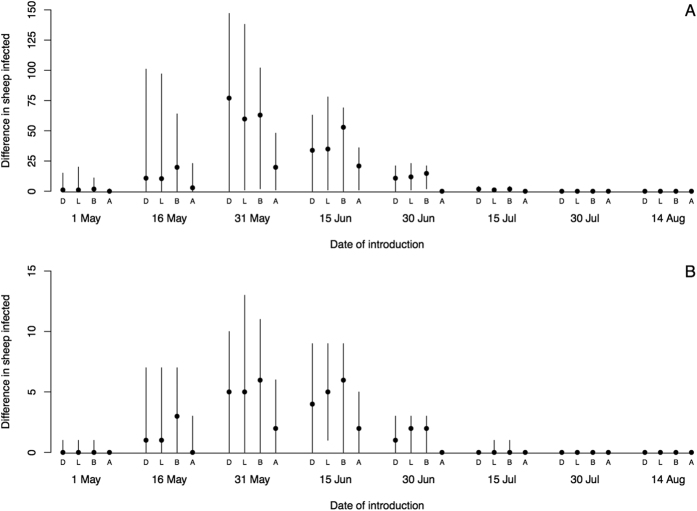
The difference between the numbers infected under a baseline scenario when (**A**) the probability of transmission from host to vector is changed from 0.01 to 0.1 and (**B**) the vector to host ratio is changed to 5,000 bites per day.

An increase in the probability of transmission from host to vector from 0.01 to 0.1 causes only a modest increase in the numbers of animals infected and after June there is no change in numbers infected (Fig. 5A). Adjusting the vector to host ratio gives similar results (Fig. 5B).

**Figure 6:**
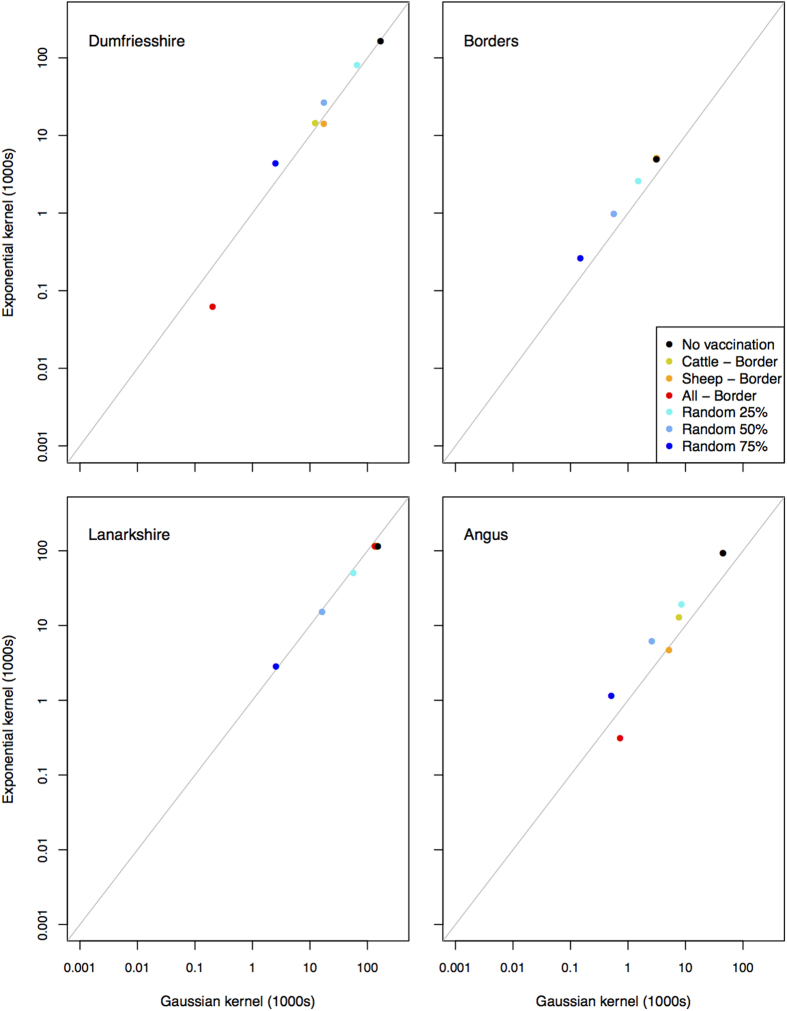
Numbers of sheep infected under the worst case scenarios for the two kernels used in these analyses.

A different transmission kernel makes a small difference to the numbers infected, but under most vaccination strategies more sheep were infected under the exponential kernel than the Gaussian kernel (Fig. 6), although the exponential kernel makes a small difference following introduction to Angus and the Borders.

## Discussion

In the event of an outbreak of BT the principal control measure that would be adopted is vaccination of susceptible livestock. However, key decisions such as which species to be vaccinated, where, and whether vaccination should be voluntary or compulsory should be underpinned by quantitative information on the relative impacts of different options. This is an instance where mathematical modelling of epidemiological emergencies under a range of scenarios can provide valuable information to policy makers in anticipation of a potential outbreak.

From the modelling conducted in this study we are able to report that under most feasible scenarios there would be minimal spread of BTv-8 in Scotland with fewer than 300 sheep infected under most scenarios. However, given an extreme case scenario in which temperatures were high and the virus proves to be well adapted to spread in cold conditions then there is very wide spread. This worst case scenario consists of:

1. Warmer (1 °C) than average temperatures.
2. A pathogen that is capable of replicating in the vector at temperatures below 13 °C.
3. An introduction during a narrow optimal time window between mid-May and mid-June.
4. An introduction to a location ideal for onward spread.

Under these scenarios the model estimates that there could be up to 105,000 sheep deaths as a consequence of BTv (Table 1).

Vaccination can provide effective control for BT, but given that it must be deployed prior to introduction of the disease, there are logistical challenges with vaccinating large numbers of animals quickly^23^. In this paper we demonstrate that for Scotland, targeting vaccination at a relatively small number of cattle in the counties bordering England delivers substantial benefits–in the worst case scenario, infection of 186,000 and deaths of 96,000 sheep can be prevented by vaccinating 518,815 cattle, but this varies by site of introduction of BT. Extending vaccination to a greater number of animals by vaccinating both cattle and sheep or by extending the spatial extent of vaccination results in a slight reduction in the number of infected animals. However, this increase in protection is at the cost of vaccinating more animals. Economic analyses of the scenarios would be needed to fully assess these implications, as well as assessment of the feasibility of vaccine implementation for larger numbers of animals.

Targeting vaccination to the species that are least affected by the disease presents serious problems for those making the decisions and may be difficult for stock keepers to accept. Whilst it is the sheep that are seriously affected by BTv-8, these results demonstrate that the most efficient way to protect the sheep in this context is to vaccinate cattle. Studies elsewhere have shown that cattle farmers may be reluctant to accept vaccine in such a scenario^24^. In the Scottish context, the cattle effectively act as the reservoir and the sheep as a spill-over host. This is further complicated by the presence of a potential wildlife reservoir in red deer which have been suggested as a species capable of sustaining BTv-8 in France^7^, but were not considered in this study.

Based on evidence of *Culicoides* feeding preferences, BTv spread in this model is driven by a reservoir in cattle. In interpreting the model outputs, we have assumed here that cattle are largely asymptomatic. However, previous studies have suggested that there may be a greater impact of BTv-8 in the cattle population which may be underrepresented here^3^,^4^. Furthermore, zooprophylaxis may be important in these contexts for minimising disease spread^25^. In a monoculture where just sheep were farmed and the same number of *Culicoides* were feeding solely on sheep then the impact of BTv-8 on sheep would be considerably greater according to the model assumptions. However, there remains the potential for further dilution of BT through vectors feeding on non-susceptible hosts, in particular horses and ponies^26^,^27^ or susceptible wildlife–in particular deer.

The impact of BT is sensitive to the site of introduction of the virus. Introduction into Dumfriesshire or Lanarkshire have substantial impact, reflecting the greater duration where R_0_ is above 1, whilst introduction into the Scottish Borders or Angus (cross border and across the North Sea) have substantially lower impact. However, the site of introduction also has a large effect on the efficacy of the strategic vaccination strategies. Timing of introduction is also important, an earlier introduction resulting in a larger epidemic. Other studies have stated that there is a higher risk of an introduction later in the year than earlier^9^. We assume that Scotland can be considered in isolation, this is largely for modelling convenience. In the event of an outbreak in southern areas, there would be an exchange of virus over the border. By modelling Scotland in isolation, virus that is in Scotland remains in Scotland rather than crossing the border. Thus, this assumption would influence the risk of introduction, it would not greatly affect the extent of spread in Scotland.

The model used here is relatively insensitive to the transmission kernel that describes the local spread. In these analyses we use two kernels–one very wide that indirectly incorporates livestock movements and another narrow kernel that is based on transmission following the introduction of a movement ban. Here we demonstrate that the impact following introduction is relatively insensitive to the selection of kernel. Introduction to new areas through simulated livestock movements often results in little BT spread due to the few areas that are suitable for BT spread. A recent study has shown that over a time period of up to three nights midges are found 1–2.5 km from their release site^28^. However, the dispersal of midges over time scales greater than two weeks that comprise the EIP of BTv in the midge has not been directly measured and as a result we used kernels derived from the spatial and temporal distribution of observed cases. The model was also insensitive to other important and uncertain parameters–the vector to host ratio and the host to vector transmission probability (Fig. 5). The vector to host ratio is highly uncertain and likely highly spatially heterogeneous^18^,^19^. In this paper we have demonstrated that under the baseline scenario in Scotland, this is robust to a range of potential parameters.

This paper has adapted a model for another vector borne disease to estimate the impact of BT in Scotland. It is a disease that is highly sensitive to parameters for spread that have not been estimated for BTv-8 and hence it was necessary to explore a range of plausible parameters, with the EIP parameters being of particular importance for determining BTv spread in Scotland. In this context it is necessary to be strategic in terms of vaccine deployment and our analysis suggests that vaccinating cattle–the species that suffer mildly relative to sheep - may be the optimal way to vaccinate. This finding was revealed by the incorporation of new data on feeding preferences in this analysis and highlights the importance of understanding vector ecology in disease transmission. In the event of an outbreak, this information could prove very valuable in targeting limited vaccine resources and identifying risks of spread. However, the role that wild ruminants such as red deer may play in acting as a reservoir for the virus in Scotland warrants further research.

## Methods and Materials

This analysis is carried out through extension of the spatially explicit stochastic model described by Bessell *et al*.^13^,^15^. This model incorporates the spatial livestock distribution from the Scottish Agricultural Census and historical temperature records. The model comprises separate compartments for host (comprising cattle and sheep) to vector transmission and vector to host transmission.

### Host to vector transmission

The duration of infection of the host was gamma distributed with a mean 16.4 days (sheep) and 20.6 days (cattle)^29^. We assume that the rate at which hosts are fed on by vectors depends on the landscape:


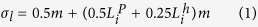


Where *m* is the baseline ratio of hosts to vectors (Table 2), where *L*^*P*^ is the proportion of pasture and grassland in a 1 km ring surrounding farm *i* and *L*^*h*^ is the proportion of heathland in the ring. This is based upon the preference for *C. obsoletus* complex for pasture and the less competent *C. impunctatus* for heathland habitat^19^,^30^,^31^,^32^,^33^. Thus, the value is 0.5 *m* if the area is not in an area with pasture, grassland or heathland, and 1 if it is surrounded by grassland or pasture.

In addition to adjusting the vector to host ratio (*m*) for the landscape, we adjusted for seasonal variations according to Searle *et al*.^10^,^18^ such that 0.25 *m* was used before 31^st^ May, between 5^th^ July and 9^th^ August and after September 18^th^, and *m* was used during the two intervening time periods.

The probability of a susceptible vector becoming infected from feeding on a viraemic host is relatively small, but estimates typically range from 0.001–0.15^31^. Due to the very large uncertainty in this parameter, we use a baseline value of 0.01 and sensitivity analysis using a value of 0.02. A third scenario is also considered with a value of 0.1.

### Vector to host transmission

The vector remains infected for a period of time that depends upon the daily extrinsic incubation rate, in this case derived from studies of BTv-9 in *C. sonorensis*^11^. Whilst this is neither a European vector species, nor the virus serotype in question here of the estimates of BT EIP that were available this was identified as the closest likely match to BTv-8 in European Palaearctic *Culicoides*^11^:


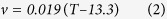


where *T* is the temperature and there is no incubation below 13.3 °C, implemented as a Poisson with mean (1/*v*)^34^. Vectors die at a daily rate given by Gerry and Mullens^35^:


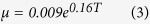


The frequency of taking blood meals is dependent on the reproduction cycle (gonotrophic cycle), given by^36^:


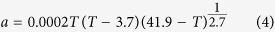


Vectors successfully transmit infection to a host with a probability of 90% based on a range of 80–100%^37^.

Vectors move between farms according to a Gaussian kernel defined by Szmaragd *et al*.^38^, a scalar is applied to this kernel to ensure that each bite is only placed once according to:


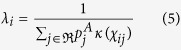


This scaling factor (*λ*) for farm *i* is given by the attractiveness of each other farm *j (p*^*A*^), which is simply the number of blood meal opportunities on that farm and the kernel distance to that farm (*κ(χ*_*ij*_)).

The Gaussian kernel implicitly incorporates animal movements, and we compare this to a kernel with no movements using an exponential kernel derived from Sedda *et al*.^39^.

Two studies have shown that when presented with a cow and a sheep, 87% of *Culicoides* will feed on the cow rather than a sheep, but they did not conclude that there are more vectors if there are cattle rather than sheep on the farm^21^,^22^. Therefore the corrected ratio of vectors to hosts is


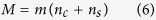


Where *m* is the ratio of vectors to each host (vector to host ratio), *n*_*c*_ is the number of cattle and *n*_*s*_ is the number of sheep on the farm.

We incorporate this on a parity basis to estimate the number of bites on cattle (m_c_) and sheep (m_s_) where *ϕ* is the likelihood that a vector will feed on a cow when presented with a cow and a sheep such that:


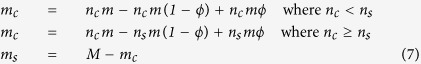


### Model implementation

The model is a stochastic simulation model with the distribution of farms and animals derived from the Scottish Agricultural Census of 2011. Mean daily temperatures were derived from UKCIP model from 1996–2011^40^ To start the stochastic process, infection was seeded on one of four selected farms based upon previously published incursion scenarios^14^. These farms were selected to represent transboundary spread from England, livestock movements and wind borne introduction over the North Sea. Infection was introduced on a number of days during the summer starting at 1^st^ May (considered day 1) and at 15 days intervals until the 14^th^ August. For each seed/start day combination, we run a number of scenarios varying certain parameters according to Table 2.

In addition, we construct a “worst case” scenario with the baseline temperature for the EIP at its lower limit, an elevated extrinsic incubation rate, temperature elevated by 1 °C and a higher host to vector transmission probability (Table 3). A further scenario with a probability of transmission from host to vector of 0.1 is also examined as well as a maximum vector to host ratio of 5,000.

### Estimating R_0_

We map the R_0_ of BTv-8 using a published model^29^, incorporating the probability of surviving the EIP from Hartemink *et al*.^41^ and informed by the parameters in this model:


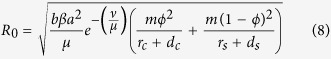


Temperature dependent parameters are fitted using long term mean temperatures for a given time of year and are expressed as the number of days in which R_0_ was greater than 1.

### Vaccination

**Figure 7:**
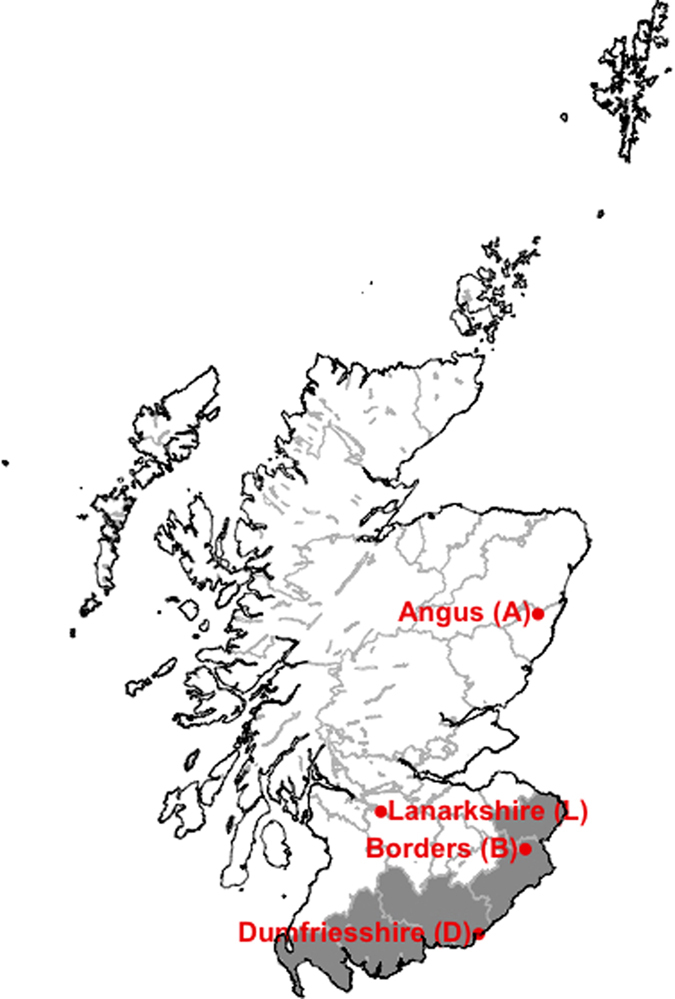
The area defined by the “border” vaccination area (darker grey) and the locations of the seeds of introduction (points). D represents short range windborne introduction or local spread from the north west of England; B represents short range windborne introduction from the north east of England; L represents an introduction by animal movements to a high risk area; A represents long range windborne introduction from north west Europe. Map created using R^45^.

The impacts of different vaccination implementation scenarios on R_0_ and disease spread were explored in this model framework. These scenarios are broadly in line with the incursion scenarios explored previously^14^. We explore vaccination of animals as either strategic compulsory vaccination or voluntary vaccination by farmer discretion. This is in line with the Defra BTv control strategy^42^, where both options are outlined. Strategic vaccination covers only those counties of Scotland that border England, and explores vaccination of cattle only, sheep only and all cattle and sheep at a given coverage that represents either imperfect vaccine efficacy or incomplete vaccine coverage (Table 4 and Fig. 7). Voluntary vaccination was assessed in the model by random allocation of a certain proportion of farms to vaccination.

### Model assumptions

There are a number of assumptions that underlie this analysis:

1. The range of dispersal of the vector can be modelled using a kernel. Vector dispersal is influenced by many factors including weather^39^,^43^. However, localised wind patterns are difficult to model in the long term and it has been demonstrated elsewhere that during periods of intense midge activity, BT transmission behaves in a similar manner to direct transmission^44^ suggesting a kernel is a suitable approximation.
2. Movements of exposed or infectious animals are not explicitly considered, although they are incorporated in the Gaussian transmission kernel^38^. In the event of an outbreak, a control zone is established within 20 km of an outbreak, a protection zone within 100 km and a surveillance zone within 150 km. Unless exemption is granted, no animals may leave this 150 km areas and animals may only move within the same zone, no movements are allowed to and from premises in the control zone^42^. A transmission kernel that incorporates these movement restrictions would resemble the exponential kernel of Sedda *et al*.^39^. However, the implementation of movement controls depends upon the timing of the detection of the first case.
3. Once infected, an animal that is not identified as having clinical signs will recover with full immunity and will not be susceptible to further infection. Those displaying clinical signs are assumed to be detected and culled once they display clinical signs.
4. The attractiveness of a farm for vector feeding is based on the number of livestock on the farm and is determined by distance to neighbouring farms and the number of livestock.
5. A proportion of animals intended for vaccination are vaccinated. However, we allow for a proportion not to be protected either representing imperfect efficacy of the vaccine or not all animals receiving the vaccine.
6. It will be logistically possible to vaccinate these populations and the vaccinated animals will develop protective immunity prior to the introduction of disease.
7. We do not incorporate vector feeding on species other than cattle and sheep. Other hosts include horses and wild ruminants, but the distribution of these species and the vector ecology in terms of feeding is relatively poorly understood.
8. Scotland can be regarded in isolation. During an epidemic involving southern Scotland there is likely to be some cross-border transmission to northern England. However, as this is likely to be a two-way exchange we consider that this would have minimal effect on the epidemic.

## Additional Information

**How to cite this article:** Bessell, P. R. *et al*. Assessing the potential for Bluetongue virus 8 to spread and vaccination strategies in Scotland. *Sci. Rep.* **6**, 38940; doi: 10.1038/srep38940 (2016).

**Publisher’s note:** Springer Nature remains neutral with regard to jurisdictional claims in published maps and institutional affiliations.

## Figures and Tables

**Table 1:** Numbers from the ‘worst-case’ scenarios of sheep mortalities (deaths due to disease and sheep culled) for different introduction sites and different timings of introduction under different vaccination scenarios.

	Number vaccinated	Intro. day	Location of introduction			
			D	L	B	A
Worst case–no vaccination	0	1	63,491	43,428	9,559	678
		16	96,560	86,588	25,738	1,785
		31	104,679	61,466	20,215	2,270
Cattle border	518,815	1	3,432	42,015	1,832	631
		16	7,096	83,335	4,443	1,802
		31	8,940	61,129	6,061	2,252
Sheep border	1,756,242	1	4,793	38,297	1,082	652
		16	10,007	79,121	2,970	1,846
		31	6,838	57,653	2,451	2,164
Cattle and sheep border	2,275,057	1	56	39,476	168	689
		16	116	76,720	418	1,789
		31	158	58,443	598	2,247
Cattle and sheep– 25% coverage	2,151,195	1	22,062	14,326	2,064	321
		16	37,502	32,476	4,845	862
		31	41,583	23,908	6,281	1,138
Cattle and sheep– 50% coverage	4,302,408	1	5,351	3,939	583	120
		16	9,988	9,282	1,479	323
		31	12,160	7,181	2,124	471
Cattle and sheep– 75% coverage	6,453,843	1	677	566	110	28
		16	1,432	1,461	293	85
		31	1,881	1,227	443	140

D represents introduction to Dumfries and Galloway, L to Lanarkshire, B to the Borders and A to Angus.

**Table 2:** Summary of parameters used in these analyses.

Parameter	Symbol	Value(s)	Comments
Ratio of vectors to hosts (baseline)	*m*	2500	Based on ref. 46 – a maximum host biting rate (*ma*) of 2500 bites per day
Spatial adjustment to *m* based upon landscape suitability for *C. obsoletus sl* and *C. puliclaris sl*	*σ*_*l*_	0.5 ≤ *σ*_*l*_ ≤ 1	Based on ref. 19
Temporal adjustment to *m* based upon seasonal variations in *Culicoides* abundance	*σ*_*t*_	[0.25, 1]	Based on ref. 18
Relative preference for feeding on cattle rather than sheep on a given farm	*ϕ*	0.87	Based upon experiments by refs 21,22 and 26
Transmission probability-vector–host	*b*	0.9	37
Transmission probability–host-	*β*	0.01	11
vector		0.02	
EIP	*1/v*	10–75 days	BTv-9. Baseline temperature [13.3, 12.7]°C and incubation rate [0.019, 0.026]^11^
Daily vector mortality rate	*μ*	0.06–0.12	35
Interval between blood meals	*1/a*	6–10	36
Duration of infection-cattle	1/r_c_	20.6	29, 47
Duration of infection–sheep	1/r_s_	16.4	29, 48
Daily probability of overt clinical signs–cattle	c_c_	0.047	Elbers^3^
Probability of overt clinical signs–sheep	c_s_	0.0218	Elbers^3^
BT induced mortality-cattle	d_c_	0	Disease induced mortality in cattle is generally very low or negligible^5^,^47^,^49^. A separate study has found low levels of mortality associated with BTv-8^3^,^4^
BT induced mortality-sheep	d_s_	0.1	3.9–14.4%^50^
Temperature adjustment		0	
		+1	
Date of introduction		1 May	Day 1
		16 May	Day 16
		31 May	Day 31
		15 June	Day 46
		30 June	Day 61
		15 July	Day 76
		30 July	Day 91
		14 August	Day 106

**Table 3:** Table of parameters comprising the different scenarios modelled in this paper.

Parameter	Scenario				
	Baseline	Increased transmission	Increased temperature	Reduced EIP	Worst case
Transmission from host to vector	0.01	0.02	0.01	0.01	0.02
Baseline EIP	13.3	13.3	13.3	12.7	12.7
EIP rate	0.19	0.19	0.19	0.26	0.26
Temperature relative to UKCIP estimate 1996–2011	Mean	Mean	Mean + 1 °C	Mean	Mean + 1 °C

**Table 4:** Vaccination scenarios tested with this model.

Scenario	Area of Scotland vaccinated	Protection coverage	Animals vaccinated
Baseline	None	None	0
Cattle border	5 counties on the English border*	90% cattle	518,815
Sheep border	5 counties on the English border*	90% sheep	1,756,242
Cattle sheep border	5 counties on the English border*	80% cattle and sheep	2,275,057
Random 25%	25% of farms vaccinate	90% protection on vaccinated farms	2,151,195
Random 50%	50% of farms vaccinate	90% protection on vaccinated farms	4,302,408
Random 75%	75% of farms vaccinate	90% protection on vaccinated farms	6,453,843

^*^Kircudbrightshire, Wigtownshire, Dumfrieshire, Roxburgh, Berwickshire.
